# Assessing the Renal Outcomes of Semaglutide in Diabetic Kidney Disease: A Systematic Review

**DOI:** 10.7759/cureus.64038

**Published:** 2024-07-07

**Authors:** Shuja ur Rehman, Nikhil Deep Kolanu, Muhammad Muaz Mushtaq, Husnain Ali, Zeeshan Ahmed, Maham Mushtaq, Maryyam Liaqat, Muhammad Asad Sarwer, Syed Faqeer Hussain Bokhari, Fazeel Ahmed, Danyal Bakht

**Affiliations:** 1 Internal Medicine, Al-Saba Hospital, Sheikhupura, PAK; 2 Internal Medicine, Allama Iqbal Teaching Hospital, Dera Ghazi Khan, PAK; 3 Internal Medicine, China Medical Univesity, Shenyang, CHN; 4 Medicine, King Edward Medical University, Lahore, PAK; 5 Internal Medicine, King Edward Medical University, Lahore, PAK; 6 Medicine, Mayo Hospital, Lahore, PAK

**Keywords:** renal protection, diabetes, acute kidney injury, albuminuria, semaglutide, diabetic kidney disease, glp-1 receptor agonist

## Abstract

Diabetic kidney disease (DKD) is a prevalent microvascular complication of diabetes, posing a significant health burden. Semaglutide, a glucagon-like peptide-1 receptor agonist, has shown promise in mitigating renal outcomes in DKD. This systematic review aimed to evaluate the renal effects of semaglutide in individuals with DKD. A comprehensive literature search identified six eligible studies, including two case reports and four cohorts, from diverse geographic locations. The primary outcomes assessed were changes in estimated glomerular filtration rate (eGFR) and albuminuria. Secondary outcomes included acute kidney injury (AKI) incidence and other renal biomarkers. The impact of semaglutide on eGFR was variable, with some studies reporting decreases and others showing improvements or no significant changes. Albuminuria, however, was more consistently reduced, particularly in patients with macroalbuminuria. Notably, the case reports described semaglutide-associated AKI, including acute interstitial nephritis, highlighting the need for careful monitoring during therapy. Beyond renal outcomes, semaglutide consistently improved glycemic control and promoted weight loss, with generally manageable gastrointestinal side effects. The findings suggest that semaglutide may effectively reduce albuminuria in DKD, potentially slowing disease progression. However, the risk of AKI and the variable impact on eGFR underscore the need for a personalized approach and vigilant monitoring, particularly in patients with advanced CKD. Future large-scale, long-term randomized controlled trials are warranted to definitively assess the renal benefits and risks of semaglutide in DKD.

## Introduction and background

Diabetic kidney disease (DKD) is a serious microvascular complication of diabetes mellitus that has emerged as a leading cause of end-stage renal disease (ESRD) worldwide [1]. Characterized by persistent albuminuria, progressive decline in glomerular filtration rate (GFR), and increased risk of cardiovascular morbidity and mortality, DKD poses a significant health burden [2]. The global prevalence of DKD is estimated to be between 20% and 40% among individuals with type 2 diabetes mellitus (T2DM), making it one of the most common diabetic complications [3]. In the United States alone, approximately 37 million adults have chronic kidney disease (CKD), with diabetes being the primary cause in about 38% of cases [4].

The impact of DKD extends beyond renal health. Patients with DKD face a substantially higher risk of cardiovascular events, hospitalization, and premature death compared to diabetic individuals without kidney involvement [5]. Moreover, the economic burden is substantial, with DKD patients incurring significantly higher healthcare costs than those without kidney disease [6]. As the global diabetes epidemic continues to grow, the prevalence of DKD is expected to rise, underscoring the urgent need for effective management strategies.

Semaglutide is a novel glucagon-like peptide-1 (GLP-1) receptor agonist that has gained attention for its potential renoprotective effects in individuals with DKD [7]. Initially developed for the treatment of T2DM, semaglutide has demonstrated efficacy in improving glycemic control, promoting weight loss, and reducing cardiovascular risk [8,9]. The mechanisms by which semaglutide might influence renal outcomes in DKD are multifaceted. First, its glucose-lowering effects may help mitigate the deleterious impacts of chronic hyperglycemia on renal function [10]. Additionally, GLP-1 receptor agonists have been shown to exert direct renoprotective effects through various mechanisms, including amelioration of inflammation, oxidative stress, and endothelial dysfunction [11,12]. Moreover, the ability of semaglutide to promote weight loss and improve metabolic parameters, such as blood pressure and lipid profiles, may indirectly contribute to the preservation of renal function [13,14].

Despite the promising potential of semaglutide in DKD, there is a need for a comprehensive systematic review to synthesize the available evidence and evaluate its efficacy and safety in this context. While several clinical trials have investigated the renal outcomes of semaglutide, the existing literature lacks a comprehensive synthesis of these findings, particularly with a focus on DKD. Gaps in the current literature include limited long-term data on the sustainability of renal benefits, the potential for differential effects in various subpopulations (e.g., based on diabetes type, age, or CKD stage), and the impact of semaglutide on hard renal endpoints, such as progression to ESRD or the need for renal replacement therapy. The specific aims of this systematic review are to evaluate the effects of semaglutide on renal function, assess its impact on the progression of DKD, and examine the potential adverse effects associated with its use in this context. By synthesizing the available evidence, this review will provide a comprehensive understanding of the renal outcomes of semaglutide in individuals with DKD.

## Review

Materials and methods

This systematic review is reported in accordance with the PRISMA (Preferred Reporting Items for Systematic Reviews and Meta-Analyses) guidelines to ensure a rigorous and comprehensive evaluation of the included studies on the renal outcomes of semaglutide in DKD.

Search Strategy

A comprehensive search was conducted in the following databases from inception to May 31, 2024: MEDLINE (via PubMed), Embase, Cochrane Central Register of Controlled Trials (CENTRAL), and Web of Science. The search strategy combined terms related to the population (e.g., "diabetic nephropathy," "diabetic kidney disease"), intervention (e.g., "semaglutide," "GLP-1 agonist"), and outcomes (e.g., "renal function," "albuminuria," "kidney failure"). Specific search terms and Medical Subject Headings (MeSH) were used to ensure inclusivity. We also performed hand-searching of the reference lists of relevant reviews and included studies to identify additional relevant publications that may not have been captured in the initial database search.

Eligibility Criteria

We included studies that met the following criteria: The population consisted of adults (≥18 years) with T2DM and DKD. The intervention involved semaglutide as monotherapy. Comparators included placebo, no treatment, or other antidiabetic agents, excluding other DPP-4 inhibitors. The primary outcomes assessed were changes in eGFR, serum creatinine, urine albumin-to-creatinine ratio (UACR), or 24-hour urine protein. Secondary outcomes included the incidence of adverse renal and non-renal events. Eligible study designs were randomized controlled trials (RCTs) and observational studies (cohort and case-control). For rare adverse events, case reports and case series were also considered. Studies involving participants without DKD or those focusing solely on glycemic control or other non-renal outcomes were excluded. We excluded animal studies, in vitro experiments, reviews, editorials, conference abstracts, and studies not published in English.

Study Selection

Two independent reviewers conducted the study selection process. Initially, titles and abstracts of all retrieved records were screened to exclude clearly irrelevant studies. Full-text articles were then obtained for all potentially eligible studies and reviewed independently by both reviewers. Any discrepancies in study inclusion were resolved through discussion, and if necessary, a third reviewer was consulted to reach a consensus.

Data Extraction

Data were extracted independently by two reviewers using a predesigned, piloted form in Microsoft Excel. Extracted information included study characteristics (author, year of publication, country, design, setting, duration), participant demographics (age, sex, baseline characteristics), intervention details (semaglutide dose, duration, co-interventions if any), and outcomes.

Data Synthesis

Given the heterogeneity in study designs, patient populations, intervention details, and outcome measures, a narrative synthesis was conducted. The findings were summarized in descriptive tables, highlighting key characteristics and results of each study. We performed a qualitative analysis to integrate and discuss the outcomes in the context of current clinical practice and future research directions. Meta-analysis was not performed due to the variability in the included studies, which precluded the pooling of results.

This comprehensive methodology ensures a rigorous, transparent evaluation of the renal outcomes of semaglutide in DKD, providing a strong evidence base to guide clinical decision-making.

Results

Study Selection Process

**Figure 1:**
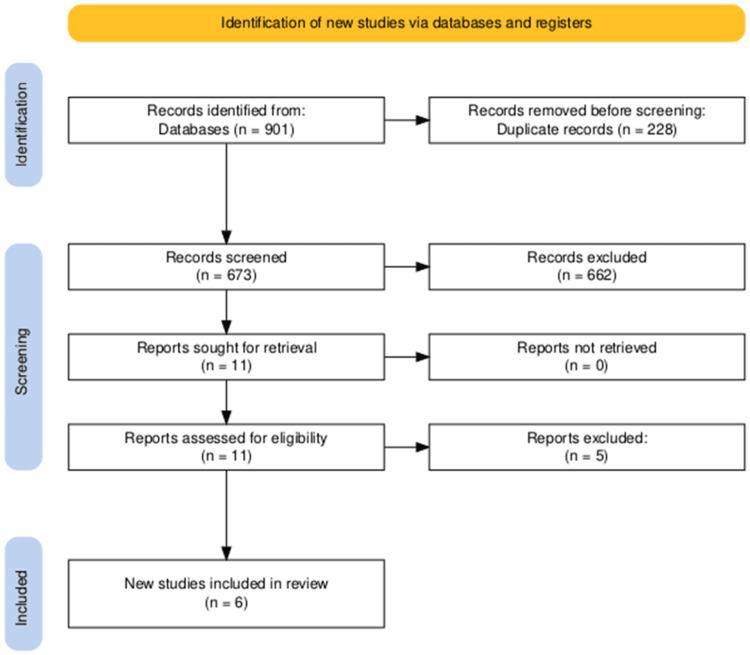
PRISMA flow diagram of selection of studies for inclusion in the systematic review. PRISMA, Preferred Reporting Items for Systematic Reviews and Meta-Analyses.

Following comprehensive database searches, 901 articles were initially identified. After removing 228 duplicates, the titles and abstracts of the remaining 673 publications were meticulously screened resulting in the exclusion of 662 articles. Subsequently, 11 potential studies underwent eligibility verification through a thorough examination of their full texts. Ultimately, six articles met the predefined inclusion criteria. The reference lists of these selected articles were also scrutinized, but no additional studies meeting the eligibility criteria were found. The entire study selection process, adhering to PRISMA guidelines, is visually depicted in the PRISMA flowchart (Figure 1).

Study Characteristics

Four observational studies (cohorts) and two case reports were included in this systematic review. One case report was from the USA and another from Canada, whereas the cohorts were from Spain, Saudi Arabia, and Japan, respectively. The studies span from 2021 to 2024, reflecting the most recent evidence in this field. The study duration of the cohorts was 12 months (Table 1).

**Table 1:** Characteristics of the studies included in this systematic review N/A: not applicable; CKD: chronic kidney disease.

Details of the study	Title					
	Acute kidney injury associated with semaglutide	Semaglutide in type 2 diabetes with chronic kidney disease at high risk progression-real-world clinical practice	Semaglutide-associated acute interstitial nephritis: a case report	Influence of chronic kidney disease and its severity on the efficacy of semaglutide in type 2 diabetes patients: a multicenter real-world study	The impact of weekly semaglutide, a glucagon-like peptide-1 agonist, on kidney outcomes in adults with type 2 diabetes mellitus	Effects of oral semaglutide on renal function in diabetic kidney disease: a short-term clinical study
Authors	Leehey et al. [15]	Aviles Bueno et al. [16]	Borkum et al. [17]	García de Lucas et al. [18]	Algarni et al. [19]	Mima et al. [20]
Publication Year	2021	2022	2022	2023	2024	2024
Journal	Kidney Medicine	Clinical Kidney Journal	Kidney Medicine	Frontiers in Endocrinology	Journal of Family Medicine and Primary Care	In Vivo
Country	USA	Spain	Canada	Spain	Saudi Arabia	Japan
Study Design	Case report	Retrospective cohort	Case report	Ambispective cohort	Retrospective cohort	Retrospective cohort
Study Duration	N/A (case reports)	12 months	N/A (single-case report)	12 months	12 months	9.0 ± 5.0 months
Sample Size	2	122	1	486 (296 no/low CKD risk, 190 CKD)	196	6

The main findings of the included studies are summarized in Table 2.

**Table 2:** Summary of the studies included in this systematic review. T2DM: type 2 diabetes mellitus; CKD: chronic kidney disease; DKD: diabetic kidney disease; eGFR: estimated glomerular filtration rate; UACR: urine albumin-to-creatinine ratio; UPCR: urine protein-to-creatinine ratio; BMI: body mass index; HbA1c: hemoglobin A1c; GLP-1RA: glucagon-like peptide-1 receptor agonist; AKI: acute kidney injury; SOB: shortness of breath; GI: gastrointestinal.

Study findings	Authors					
	Leehey et al. [15]	Aviles Bueno et al. [16]	Borkum et al. [17]	García de Lucas et al. [18]	Algarni et al. [19]	Mima et al. [20]
Population	T2DM with CKD due to DKD	Patients with T2DM and CKD (eGFR <60 mL/min/ 1.73 m^2^) and/or UACR >30 mg/g	Male in his thirties with morbid obesity, diabetes, non-ischemic cardiomyopathy	T2DM patients, with or without CKD	Adults aged 18 and above with T2DM who received weekly subcutaneous semaglutide for at least six months	Patients with DKD
Age	Early 80s (Case 1), 60s (Case 2)	65.50 ± 11 years	30s	CKD group: 66.6, No/low CKD: 58.4	Mean age = 65.38 years (range: 42-85 years)	69.2 ± 7.9 years
Sex	Female (Case 1), male (Case 2)	62% males, 38% females	Male	CKD group: 57.9% males, 42.1% females, no/low CKD risk: 47% males, 53% females	58.7% males, 41.3% females	4 males, 2 females
Baseline Characteristics	Case 1: eGFR: 11 mL/min/1.73 m^2^, UPCR: 4.9 g/g. Case 2: eGFR: 22 mL/min/1.73 m^2^, UPCR: 1.33 g/g	Mean weight: 98.72 kg, mean HbA1c: 7.57%, mean eGFR: 50.32 mL/min/1.73 m^²^, mean BMI: 35.8 ± 4.79 kg/m^2^. 74% had micro- or macroalbuminuria	Serum creatinine: 12.86 mg/dL, urinary albumin-creatinine ratio: 91.1 mg/g, HbA1c: 7.7%	HbA1c: CKD: 8.75%, no/low CKD: 8.30%, body weight: CKD: 98.5 kg, no/low CKD: 98.9 kg, BMI: CKD: 36.4 kg/m², no/low CKD: 36.5 kg/m², eGFR: CKD: 49.5 mL/min/1.73 m², no/low CKD: 89.79 mL/min/1.73 m², UACR: CKD: 58.00 mg/g, no/low CKD: 6.00 mg/g	Mean weight: 86.69 kg, mean BMI: 33.07 kg/m², mean HbA1c: 9.18, mean eGFR: 48.07	BMI: 27.8 kg/m^2^, Δ eGFR: -1.2 mL/min/1.73 m², Δ urinary protein-creatinine ratio: −0.6 ± 2.4, duration of diabetes: 12.3 ± 10.0 years
Intervention Type	Subcutaneous semaglutide	Subcutaneous semaglutide	Subcutaneous semaglutide	Subcutaneous semaglutide	Subcutaneous semaglutide	Oral semaglutide
Dosage	Case 1: 0.25-0.5 mg weekly, Case 2: 0.25-0.75 mg weekly	Starting at 0.25 mg, increasing to 0.5 mg, then 1 mg	0.25 mg, increased to 0.5 mg	1 mg (target dose)	0.25-1 mg (mean = 0.75 mg)	3 mg/day
Frequency	Once weekly	Once weekly	Once weekly	Once weekly	Once weekly	Once daily
Primary Renal Outcomes						
eGFR Change	Case 1: Decreased from 30 to 11, Case 2: decreased from 30-35 to 22	Small insignificant increase of 2.2 mL/min/1.73 m²	Decreased from 91 to requiring dialysis, then recovered to baseline	No significant improvement in any group	Slight decrease (1.35 units on average), not statistically significant (p = 0.059)	Δ eGFR: 0.7 ± 1.8 mL/min/1.73 m^2^ at 6 months
Albuminuria	Case 1: Increased from <1 to 4.9 g/g, Case 2: increased from 0.4-0.5 to 1.33 g/g	51% decrease in UACR at 12 months in macroalbuminuria group	UACR Increased from undetectable to 91.1 mg/g, then recovered to <8.84 mg/g	Noticeable improvement in UACR through the 12 months of treatment was observed, especially in the first six months	Statistically significant reduction in UACR values from median 5.97 pretreatment to 5.60 post-treatment (p = 0.005)	No significant change in proteinuria
Secondary Renal Outcomes						
AKI Incidence	Both cases developed acute kidney injury	Not reported	Semaglutide-associated acute interstitial nephritis	Not reported	Not reported	Not reported
Other Renal Biomarkers	Not reported	Not reported	Serum creatinine increased from 1.05 to 12.86 mg/dL, then recovered to 1.32 mg/dL	Not reported	Not reported	Not reported
Adverse Events	Case 1: Nausea, vomiting, Case 2: decreased appetite, fatigue, weight loss	5.7% stopped semaglutide, mostly for digestive intolerance, but also due to weight loss failure	SOB, malaise	Mostly transient GI events, few severe hypoglycemic episodes, one urinary tract infection	None reported	None reported
Main Findings	These case reports describe two patients with type 2 diabetes and chronic kidney disease who experienced acute kidney injury and worsening proteinuria shortly after initiating semaglutide, a GLP-1 receptor agonist. The authors recommend caution with using these agents in patients with moderate to severe CKD.	Patients with T2DM and CKD treated with subcutaneous semaglutide for 12 months showed significant improvements in glycemic control and weight loss. Albuminuria decreased by over 50% in those with macroalbuminuria. GLP-1RA treatment was safe and well tolerated.	This case report describes the first known instance of semaglutide-induced acute interstitial nephritis in a patient without pre-existing chronic kidney disease. The authors suggest that as GLP-1 receptor agonist prescriptions increase, particularly in CKD patients, regular clinical follow-up and careful kidney function monitoring during dose escalation should be considered.	The study found that semaglutide effectively improved glycemic control and reduced weight in type 2 diabetes patients, regardless of CKD status. Weight loss was slightly better in non-CKD patients. Notably, semaglutide significantly reduced albuminuria, especially in severe CKD, and improved CKD status in 30-40% of patients, with no major safety concerns.	Semaglutide improved weight loss, glycemic control, reduced UACR, and a reduced risk of nephropathy, showing potential renal benefits for T2DM patients.	Despite the small sample size and short observation period, oral semaglutide was found to be a relatively well-tolerated drug that tended to improve eGFR in patients with diabetic kidney disease, but did not reduce proteinuria.

Discussion

Our review encompassed six studies, ranging from case reports to retrospective cohort studies, conducted across diverse geographic locations including the United States, Spain, Canada, Saudi Arabia, and Japan. This geographical diversity enhances the generalizability of our findings. The studies collectively involved over 800 participants, primarily focusing on T2DM patients with varying degrees of CKD, from those at low risk to those with severe impairment. The primary renal outcomes examined were changes in estimated glomerular filtration rate (eGFR) and albuminuria, both critical markers of kidney function and damage in DKD. Secondary outcomes included the incidence of acute kidney injury (AKI) and changes in other renal biomarkers. The intervention across all studies was semaglutide, administered either subcutaneously (once weekly) or orally (daily), with dosages ranging from 0.25 mg to 1 mg for subcutaneous administration and 3 mg/day for oral administration.

Interestingly, the impact of semaglutide on eGFR was variable across studies. Leehey et al. observed significant eGFR decreases in both patients following semaglutide initiation, from 30 to 11 mL/min/1.73 m² in one case and 30-35 to 22 mL/min/1.73 m² in another [15]. In contrast, Aviles Bueno et al. noted a small, insignificant increase of 2.2 mL/min/1.73 m² [16]. Similarly, Mima et al. reported a slight improvement in eGFR of 0.7 ± 1.8 mL/min/1.73 m² at six months [20]. Algarni et al. observed a slight, non-significant decrease (1.35 units on average, p = 0.059) [19]. These disparities suggest that the effect of semaglutide on eGFR may be patient-specific, possibly influenced by factors such as baseline kidney function, diabetes duration, and comorbidities.

The impact on albuminuria, a key marker of glomerular damage, was more consistently positive. Aviles Bueno et al. reported a substantial 51% decrease in UACR at 12 months in patients with macroalbuminuria [16]. Similarly, García de Lucas et al. observed noticeable improvements in UACR over 12 months, particularly in the first six months [18]. This finding was echoed by Algarni et al., who reported a statistically significant reduction in UACR from a median of 5.97 to 5.60 (p = 0.005) [19]. These results suggest that semaglutide may effectively reduce albuminuria in DKD patients, potentially slowing disease progression. However, it is crucial to note that not all studies showed this benefit. Leehey et al. described increased proteinuria following semaglutide initiation, from <1 to 4.9 g/g in one case and 0.4-0.5 to 1.33 g/g in another [15]. Similarly, Mima et al. found no significant change in proteinuria [20]. These contrasting findings underline the complexity of DKD pathophysiology and the heterogeneity in patient responses to GLP-1 receptor agonists.

A significant concern raised by our review is the potential for semaglutide-induced AKI. Both patients reported by Leehey et al. developed AKI shortly after starting semaglutide [15]. Borkum et al. documented the first known instance of semaglutide-associated acute interstitial nephritis [17]. The patient, a male in his thirties without pre-existing CKD, experienced a dramatic increase in serum creatinine from 1.05 to 12.86 mg/dL, necessitating hemodialysis. Although kidney function eventually recovered, this case underscores the need for vigilant monitoring during semaglutide therapy, particularly during dose escalation.

Beyond renal outcomes, the studies consistently reported improvements in glycemic control and substantial weight loss with semaglutide. García de Lucas et al. found that semaglutide effectively lowered HbA1c and body weight regardless of CKD status, with slightly better weight loss in non-CKD patients [18]. Algarni et al. also noted significant improvements in these parameters. These benefits are particularly relevant in DKD, where obesity and poor glycemic control are major risk factors for disease progression [19]. Adverse events were predominantly gastrointestinal, including nausea, vomiting, and decreased appetite. In the study by Aviles Bueno et al., 5.7% of patients discontinued semaglutide, mostly due to digestive intolerance [16]. While these side effects are generally manageable, they highlight the importance of patient education and gradual dose titration to enhance tolerability.

The strengths of our review lie in its comprehensive approach, incorporating diverse study types from various global regions. This breadth provides a more nuanced understanding of the renal effects of semaglutide in different patient populations. However, there are notable limitations. Most studies were observational, lacking the robust design of RCTs. Sample sizes were often small, particularly in case reports and short-term studies like Mima et al. [20]. Follow-up periods were generally short, which may not capture the long-term renal effects of semaglutide. Additionally, the studies varied in their patient populations, semaglutide formulations (subcutaneous vs. oral), and dosages, complicating direct comparisons. Future research should prioritize large-scale, long-term RCTs to definitively assess the renal benefits of semaglutide as well as its risks in DKD. Studies should stratify patients by CKD stage to elucidate whether certain subgroups derive greater benefits or face higher risks. Comparative studies with other GLP-1 receptor agonists and novel agents like sodium-glucose cotransporter-2 inhibitors would also be valuable. Additionally, mechanistic studies are needed to understand how semaglutide affects kidney function at the molecular level, particularly in the context of AKI. In the interim, clinicians should weigh the potential benefits of glycemic control and albuminuria reduction against the risk of AKI when considering semaglutide for DKD patients. A personalized approach, taking into account individual patient characteristics and closely monitoring renal function, is advisable. As our understanding evolves, semaglutide may become a key player in the multimodal management of DKD, offering hope to millions grappling with this challenging condition.

## Conclusions

This systematic review highlights the potential renoprotective effects of semaglutide in DKD, particularly its ability to reduce albuminuria, a key marker of glomerular damage. However, the impact on the eGFR remains variable, and the risk of AKI, including acute interstitial nephritis, is a significant concern. Clinicians should carefully weigh the potential benefits against the risks when considering semaglutide for DKD patients, adopting a personalized approach, and closely monitoring renal function. Future large-scale, long-term RCTs are crucial to definitively establish the renal efficacy and safety profile of semaglutide in DKD patients, stratified by disease severity. As our understanding evolves, semaglutide may become a valuable therapeutic option in the multimodal management of this challenging condition.
